# Anonymous online cognitive behavioral therapy for sleep disorders in shift workers—a study protocol for a randomized controlled trial

**DOI:** 10.1186/s13063-021-05437-9

**Published:** 2021-08-16

**Authors:** Lukas Retzer, Monika Feil, Richard Reindl, Kneginja Richter, Robert Lehmann, Mark Stemmler, Elmar Graessel

**Affiliations:** 1 Faculty for Social Sciences, University of Applied Sciences Nuremberg Georg-Simon-Ohm, Nuremberg, Germany; 2 University Clinic for Psychiatry and Psychotherapy, Paracelsus Medical University Nuremberg, Nuremberg, Germany; 3 Department of Psychology, Friedrich-Alexander University Erlangen Nuremberg, Nuremberg, Germany; 4 Department of Medical Psychology and Medical Sociology, University Clinic for Psychiatry and Psychotherapy, Erlangen University Hospital, Friedrich-Alexander University Erlangen Nuremberg, Erlangen, Germany

**Keywords:** Cognitive behavioral therapy, E-Health, Shift work, Intervention studies, Circadian rhythm sleep disorder, Insomnia, Study protocol

## Abstract

**Background:**

Many shift workers suffer from sleep issues, which negatively affect quality of life and performance. Scientifically evaluated, structured programs for prevention and treatment are scarce. We developed an anonymous online cognitive behavioral therapy for insomnia (CBT-I) program. After successful completion of a feasibility study, we now start this prospective, randomized, controlled superiority trial to compare outcomes of two parallel groups, namely an intervention group and a waiting-list control-group. Additionally, we will compare these outcomes to those of a face-to-face CBT-I outpatient sample.

**Methods:**

Collaborating companies will offer our anonymous online intervention to their shift-working employees. Company physicians and counseling services will screen those interested for inclusion and exclusion criteria. Participants will receive access to our online service, where they will complete psychometric assessment and receive random assignment to either the intervention group or the waiting-list control group. Participants and providers will be aware of the group assignment. We aim to allocate at least *N* = 60 participants to the trial. The intervention consists of psychoeducation, sleep restriction, stimulus control, relaxation techniques, and individual feedback delivered via four e-mail contacts. During the intervention, as well as during the waiting period, participants will fill out weekly sleep diaries. Immediately after completion of the program, the post-intervention assessment takes place. Participants in the control group will be able to participate in the program after all study assessments. To recruit an additional sample, collaborating outpatient sleep clinics will provide six sessions of standard face-to-face CBT-I to an ad hoc sample of shift working patients. We expect both the online and the face-to-face CBT-I interventions to have beneficial effects compared to the control group on the following primary outcomes: self-reported symptoms of depression and insomnia, sleep quality, and daytime sleepiness.

**Conclusions:**

The online intervention allows shift workers to follow a CBT-I program independently of their working schedule and location. Forthcoming results might contribute to further improvement of prevention and therapy of sleep issues in shift workers.

**Trial registration:**

German Clinical Trials Register DRKS DRKS00017777. Registered on 14 January 2020—retrospectively registered.

## Trial status

Protocol version: v1.1, 01/12/2021

The study began recruitment on 11/01/2019. We plan to complete recruitment by 05/31/2022.

## Introduction

While working during the day is still the standard in western society, about one in six workers is on a shift schedule [1, 2], a proportion that has remained largely constant since 1985 [3]. Shift work is associated with various health complaints, including cardiovascular, gastroenterological, and oncological diseases, as well as sleep disorders like insomnia and circadian rhythm sleep disorder [4–7]. Factors that cause sleep issues in shift workers include psychosocial stress [7, 8], negative health behaviors [9], and circadian misalignment [10, 11]. Approximately one in three shift workers suffers from at least one clinically relevant sleep disorder, most often closely related to the individual shift schedule [10, 12–14]. If such a disorder persists for more than 3 months, the International Classification of Sleep Disorders [15] refers to it as “shift work sleep disorder” (SWSD). There exists some evidence on how to prevent SWSD by ergonomic redesign of work environments, mostly focused on the topics of bright artificial light during night shifts, nap breaks, and changes in shift rotation [16]. However, formulating clear guidelines presents a challenge to researchers because of conflicting evidence on most of these topics. For manifest SWSD, treatment options described in the literature are almost always limited to pharmacological approaches, most prominently including the use of melatonin [17–19].

The standard treatment of sleep disorders not related to shift work is cognitive behavioral therapy for insomnia (CBT-I) [20, 21], which has consistently shown sustained moderate to large positive effects [22]. CBT-I has also been successfully applied in shift workers in two published studies [23, 24], where Järnefelt and colleagues have shown the potential for group delivered CBT-I interventions in participants who worked irregular hours and suffered from insomnia or SWSD. However, we argue that many shift workers might not be able to participate in regularly scheduled group or even one-on-one meetings due to their working hours. For the treatment of shift workers, we thus argue for a transfer of CBT-I contents from synchronous face-to-face to asynchronous online modes of communication, which do not rely on simultaneous presence of provider and recipient [25]. Multiple studies have shown the transfer of CBT-I contents to online settings to be feasible and effective in reducing insomnia symptoms, both in non-shift workers [26–28] and shift workers [29]. Improvements in sleep tracking and dropout rate management, as well as more validation studies and expanded public access, are needed [30, 31].

Our group conducted a non-randomized feasibility and non-inferiority trial on the effects of an online CBT-I intervention (*n* = 22) compared to a face-to-face setting (*n* = 20) [29]. We were able to show significant improvements regarding symptoms of insomnia and depression in both settings. Contents of the program are rooted in the CBT-I literature [32, 33] and have largely remained the same for this study. Based on the findings in the feasibility study, we adjusted the online platform and the study design in order to reduce the dropout rate and to increase the validity and transferability of the results. We will now begin to evaluate the efficacy of the online CBT-I intervention in a randomized controlled trial. In this manuscript, we present a description of the updated study protocol.

The purpose of this study is to evaluate the effects of online CBT-I in shift workers, compared to a waiting-list control group and face-to-face CBT-I. Specifically, we hypothesize both CBT-I interventions to be associated with greater improvements than the control condition regarding the following outcomes: sleep efficiency as reported by the participants in sleep diaries [34]; symptoms of depression as measured by the Beck Depression Inventory (BDI-II) [35]; symptoms of insomnia as measured by the Regensburg Insomnia Scale (RIS) [36]; and daytime sleepiness as measured by the Epworth Sleepiness Scale (ESS) [37].

## Methods

This prospective, controlled, superiority trial compares outcomes of two parallel groups, namely an online intervention group and a waiting-list control group, randomly assigned in a 1:1 ratio, and a third, non-randomized ad hoc sample, namely a face-to-face intervention group.

Data for the online intervention group and the control group will be collected at the Institute for E-Counselling, University of Applied Sciences Nuremberg Georg-Simon-Ohm, Germany. The Institute is a department of the Faculty for Social Sciences. It is specialized in developing, implementing, and evaluating online counseling solutions. The partnering companies that will offer the online intervention to their employees include multiple plants of a large German engineering and technology company and two German municipal hospitals. Data for the face-to-face intervention group will be collected at the outpatient clinic for sleep medicine at the University Clinic for Psychiatry and Psychotherapy, Paracelsus Medical University Nuremberg, Germany, as well as partnering outpatient sleep clinics across Germany.

For the online conditions, participants are included starting in November 2019 and ending in May 2022, according to the eligibility criteria listed in Table 1. Prior to granting access to the online platform, company physicians or social counseling services will obtain informed consent. Participants will be anonymous to the CBT-I professional.

**Table 1:** Eligibility criteria for participants

Inclusion criteria	
Age ≥ 18	
Regularly (> 6 times per month) working outside the hours of 6 a.m. to 10 p.m.	
Subjective sleep issues diagnosed in the screening process	
Exclusion criteria	
Acute suicidality as measured by the respective item in the BDI-II at T0	
Ongoing alcohol or drug abuse	
No access to required technology (PC, smartphone, tablet)^a^	

^a^Only applies to online conditions

For the face-to-face condition, collaborating clinics will include participants from August 2020 to May 2022. Most of the same eligibility criteria apply (see Table 1).

Both the online and the face-to-face intervention are based on CBT-I principles [32]. The online program consists of psychoeducation, sleep restriction, and stimulus control, all delivered via personalized e-mails, as well as relaxation techniques, delivered via text and audio files. The face-to-face intervention consists of the same contents, delivered in 45-min, one-on-one outpatient sessions. Psychoeducation includes presenting general information about sleep and sleep disorders in order to challenge misperceptions and faulty beliefs about insomnia and its consequences as well as sleep hygiene education. In sleep restriction, the CBT-I professional instructs participants to reduce their time in bed and adhere to regular bedtimes. This creates a mild state of sleep deprivation, ultimately increasing sleep quality and decreasing fragmentation of sleep. While following stimulus control, participants use their beds exclusively for sleep in an attempt to disassociate the bed with potentially stressful or exciting activities. This entails leaving the bed and the bedroom if wakefulness persists for more than 15 min and only returning to bed once sufficient sleepiness has set in. Relaxation techniques include breathing exercises and progressive muscle relaxation. Some parts of the program have been adjusted to better fit the needs of shift workers. Most importantly, we will not recommend strictly regular bedtimes, often referred to as sleep windows [32], and instead work with anchor sleep [38]. In this approach, participants are encouraged to find the longest possible overlap between the times they spend in bed during each 24-h cycle, irrespective of the specific shifts they work. For more detailed descriptions of the CBT-I contents, we refer to our feasibility study [29] and the primary literature [32].

After registration, T0 measurements and randomized allocation, participants in both of the online groups will receive access to the first weekly sleep diary along with instructions on how to proceed. Every time participants in the online intervention group complete a week of diary entries, they will receive the next e-mail with individual feedback and further CBT-I contents. In the face-to-face intervention, participants will fill out sleep diaries between sessions. We will encourage participants to leave as few nights undocumented as possible; however, breaks in the sleep protocols will be permitted. Following our experience from the feasibility study, we expect that participants complete the program within 6 to 12 weeks. We will consider an intervention complete after a participant has received all CBT-I contents and filled out at least 4 weeks of sleep diaries.

T0 will include screening questions for sleep issues such as periodic limb movement disorder, sleep apnea, and sleepwalking, as well as questions regarding concurrent and previous treatments. Changes in medication will be reported in the sleep diaries. We will not exclude participants from the trial based on these questions; however, we might recommend alternative courses of treatment and we will control for relevant changes in treatment or medication during the trial when analyzing the data.

Outcome measures of the study will include data from at least four sleep diaries (total sleep time and time in bed for each night, average sleep efficiency across seven consecutive nights) as well as psychometric self-report scales at T0 and T1. The BDI-II is a 21-item self-report measure for depressive symptoms [35]. The RIS is a 10-item self-report questionnaire for cognitive, emotional, and behavioral aspects of psychophysiological insomnia [36]. The ESS is an 8-item self-report scale for daytime sleepiness [37]. In addition, T0 will include the 15-item self-report Morningness-Eveningness-Stability-Scale improved (MESSi) [39], from which the individual chronotype will be calculated, as well as three screening questions for sleep apnea, snoring, and periodic limb movement disorder. This will be used to guide the CBT-I treatment and explore potential differences in treatment response [40].

**Fig. 1:**
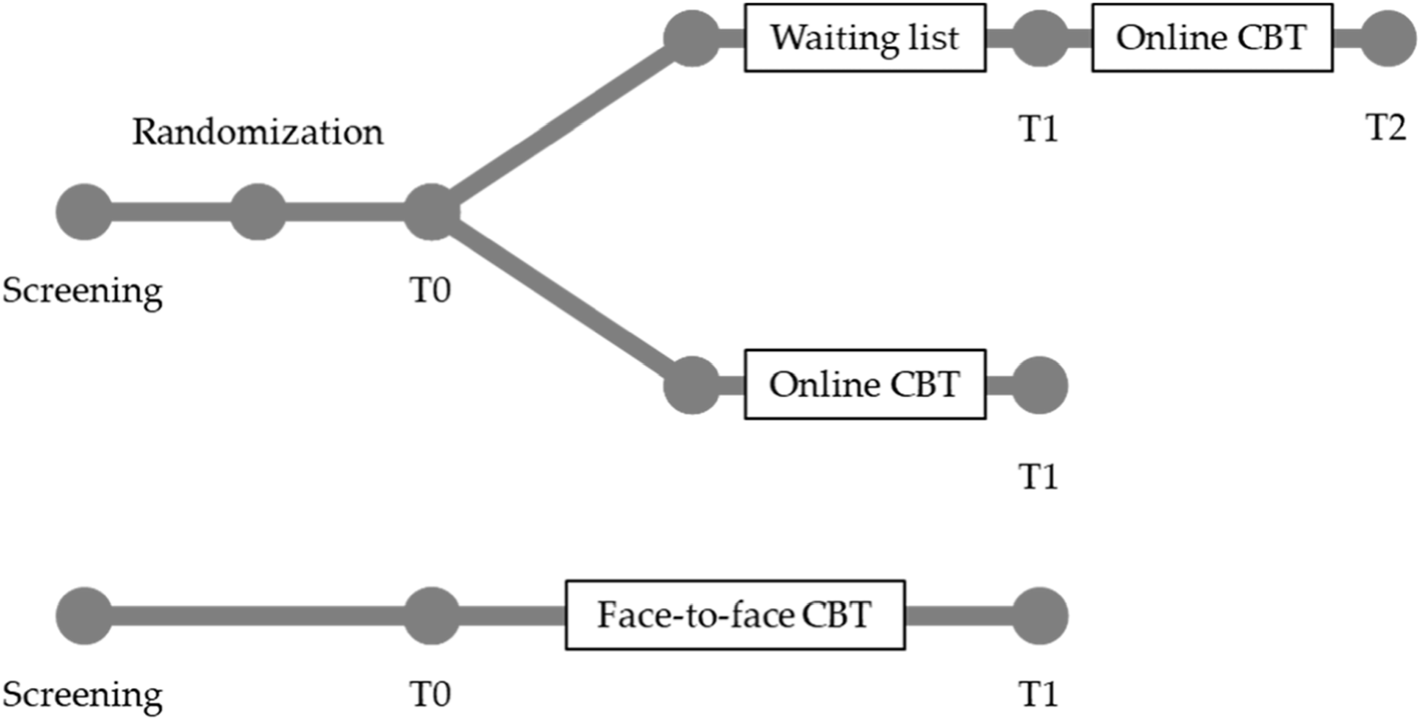
Study design

Figure 1 and Table 2 illustrate the design of the study. For the online arms of the trial, company physicians and social counseling services in collaborating companies will screen those interested for eligibility. We describe inclusion and exclusion criteria in Table 1. Psychometric assessments are carried out on the online platform after logging on for the first time (T0) and after filling out the last of four weekly sleep diaries (T1). Immediately after T0, participants are allocated randomly in a 1:1 ratio to the intervention or the control group. The intervention group will receive the CBT-I contents via e-mail between their weekly sleep diaries (W1 through W4). After completing T1, participants in the waiting group will be able to follow the CBT-I program. If they choose to do so, they will complete at least four more sleep diaries during the CBT-I period and we will contact them for a follow-up (T2) after completion. For the face-to-face arm, psychologists and CBT-I professionals in collaborating outpatient sleep clinics will screen their patients for eligibility according to the criteria in Table 1. Psychometric assessments are carried out right before the first (T0) and right after the last session (T1) (see Table 2).

**Table 2:**
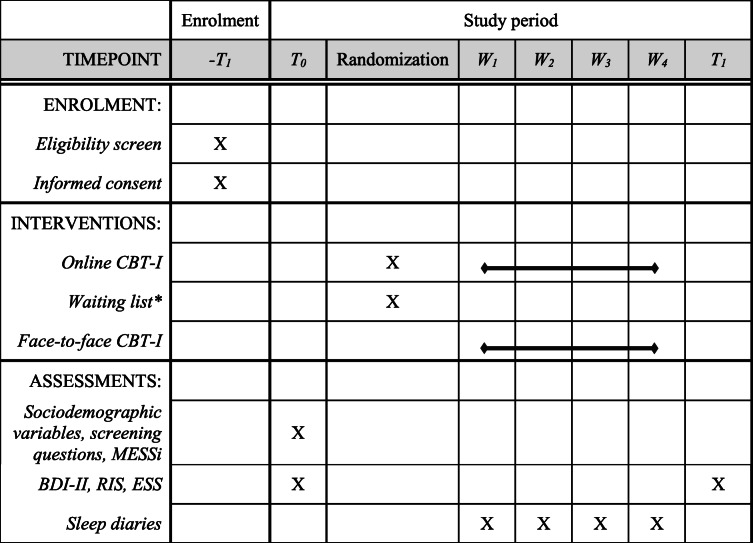
Schedule of enrolment, interventions, and assessments as recommended by the Standard Protocol Items: Recommendations for Interventional Trials (SPIRIT) statement [41]

*Optional CBT-I and T2 after waiting list not shown here in the interest of readability

The effect size for the CBT-I treatment in our feasibility study [29] (*n* = 22) was *d* = 0.76, corresponding to *f* = 0.38. The correlation among the repeated measures reached a value of *r* = .67. Setting the *α*-error probability at .05, *β*-error probability at .20, the number of groups at 3, and the number of measurements at 2, we calculated a required sample size of *N* = 15 using the ANOVA repeated measures, within-between interaction setting of the G*Power software [42]. If we adjust the effect size to a more conservative *f* = 0.25, the required sample size is *N* = 30. Consistent with this magnitude, Bortz and Schuster [43] recommend a sample size of at least *N* = 19 for large effect sizes, high power values, and low α-error probabilities. Expecting a maximum dropout rate of 50%, as observed in the feasibility study, we aim to allocate 60 participants and evaluate at least 30 participants (10 per group). We will make an effort to obtain assessments for participants who drop out of the study to facilitate carrying out intention-to-treat analyses.

Company physicians and social counseling services at collaborating companies will inform their workforce about the trial and screen those interested for eligibility. They will use flyers and posters to reach as many potential participants as possible, for example on company health days. They will also be the ones to obtain informed consent before granting access to our anonymous online platform. No personal information about potential or enrolled trial participants will be shared with the investigators. Vice versa, investigators will not share personal information with the company physicians and social counseling services. Exceptions to this rule are groups of at least ten participants, where sample characteristics, such as the average rate of improvement, may be communicated to the enrolling services in collaborating companies. No such data will be shared if inferences to individual cases are possible at any level or if any subsample of interest is smaller than ten.

Group assignment takes place immediately after receiving T0 measurements. The CBT-I professionals will assign their respective participants to the intervention and the waiting group in a 1:1 ratio using an Excel sheet, where every anonymous, randomly assigned access code has been randomly assigned a value of 0 or 1, corresponding to the waiting list and the intervention group respectively. Those responsible for data evaluation will be blinded regarding group assignments. Participants and providers will be aware of the assignment.

Participants will enter their baseline (T0) and outcome (T1) data described under Outcomes, as well as their sleep diaries through our online platform and transmit it to the Institute’s servers. We will contact participants that dropped out or otherwise failed to transmit four sleep diaries for T1 measurements.

Data will be exported from our servers and entered into SPSS [44] via the program’s syntax function. The raw data will remain on the servers for storage security. We will check the data for errors resulting in outliers or missing values. Missing values will not be replaced. Dropouts will be analyzed separately Group comparisons and superiority tests will be computed in SPSS using repeated measures ANOVA.

A data monitoring committee will not be needed, as the CBT-I professionals are not blinded during the trial and can react to any adverse events. The sleep restriction element of the CBT-I intervention is the only part of the trial where adverse events are expected to occur based on experiences from outpatient treatment with the same contents. If a participant reports issues in the form of a critical drop in total sleep time (< 4h for 7 nights or more) with this part of the treatment, CBT-I professionals are instructed to try to use a less strict approach. If this is still not tolerated well enough, we will stop sleep restriction and proceed with the rest of the contents. This should result in a quick recovery. Reports of adverse events will be collected alongside any other solicited or spontaneously reported feedback from participants. Interim analyses, trial conduct audits, and stopping guidelines are not intended at this point.

The study was approved by the ethics committee of the Lutheran University of Applied Sciences Nuremberg, Germany, under the trial ID am/sot. Changes to the protocol will be communicated to the sponsors, trials registry, and ethics committee within 6 months of their implementation.

The authors declare that there are no financial or other competing interests for the trial. Only the coordinating investigators LR and MF will have access to the final trial dataset. There are no publication restrictions. The results of this trial will be submitted to publication within 12 months of the end of the trial. The same authors who are responsible for this study protocol will be eligible for authorship of the final publication. No professional writers will be employed.

## Discussion

The prevalence of sleep disorders among shift workers is high, yet evidence-based programs for prevention and treatment of these problems in this target population are scarce. Providing a low threshold to treatment by transferring CBT-I contents to an online platform and testing the efficacy with a randomized design seems profitable for potential participants as well as our field of study.

One of the limitations of this design is the lack of a scheduled follow-up measurement 6 to 12 months after T1. If we find positive results for our treatment, we will not be able to attest to the sustainability of these results. However, in order to contact former participants after the trial, we would have to collect personal information such as phone numbers or email addresses, which would be in direct conflict with our promise of an explicitly anonymous platform. Future studies could address the long-term effects of online CBT-I. Another limitation concerns the non-randomized allocation between the face-to-face and the online settings. We will thus have to remain mindful of potential confounding factors and remain very cautious in comparing online and face-to-face group outcomes. There are multiple reasons as to why we chose to allocate participants in this manner. Firstly, collaborating companies are distributed across all of Germany, making it impossible for some of the potential participants to realistically reach one of the outpatient clinics for regularly scheduled appointments. Secondly, we promise anonymous treatment on our online platform, which we could not offer in a face-to-face setting.

We hope that the results of this study contribute to the growing evidence on the efficacy of online CBT in general and online CBT-I in shift workers in particular. If effective, our intervention offers a low-threshold, cost-effective, and readily transferrable approach to treatment and prevention of sleep disorders in the shift working population that decreases symptoms of depression and insomnia and improves daytime sleepiness.

## Data Availability

Only the coordinating investigators LR and MF will have access to the final trial dataset. There are no contractual data access restrictions.
